# Investigation on the Efficacy of Two Food Supplements Containing a Fixed Combination of Selected Probiotics and β-Glucans or Elderberry Extract for the Immune System: Modulation on Cytokines Expression in Human THP-1 and PBMC

**DOI:** 10.3390/foods13030458

**Published:** 2024-02-01

**Authors:** Giorgio Cappellucci, Giulia Baini, Elisabetta Miraldi, Lara Pauletto, Heide De Togni, Floriana Raso, Marco Biagi

**Affiliations:** 1 Department of Physics, Earth and Environmental Sciences, University of Siena, 53100 Siena, Italy; cappellucci@student.unisi.it (G.C.); giulia.baini2@unisi.it (G.B.); elisabetta.miraldi@unisi.it (E.M.); 2 Scientific Affairs Department Schwabe Pharma Italia, 39044 Egna, Italy; lara.pauletto@schwabe.it (L.P.); heide.detogni@schwabe.it (H.D.T.); floriana.raso@schwabe.it (F.R.); 3 Department of Food and Drug, University of Parma, 43124 Parma, Italy

**Keywords:** probiotics, beta-glucans, herbal extracts, immune system, food supplements

## Abstract

Several herbal and other natural products are used as ingredients in food supplements to strengthen immunity even if, very often, marketed products are proposed without a clear rationale or experimental evidence. In this study, we aimed to investigate the effect on human monocytes (THP-1) and on ex vivo human peripheral blood mononuclear cells (PBMC) of two formulations, one containing *Bifidobacterium animalis* subsp*. lactis* Bl-04^®^ with β-glucans (for adults) and one containing *Lactobacillus rhamnosus* CRL1505 with elderberry extract (for children). We compared formulations with single ingredients, with bacterial lipopolysaccharide (LPS) and the drug pidotimod; cytokines expression level was evaluated testing different concentrations of samples at two exposure times. As expected, LPS caused a non-specific huge upregulation of cytokines expression both in THP-1 and in PBMC, whereas pidotimod mainly upregulated IL-2 in PBMC and IL-8 in THP-1. The two formulations showed a difference between a pro-inflammatory stimulus such as LPS, and also from an immunostimulant drug, such as pidotimod, as they mainly upregulated the expression of IL-6 and IL-10 in PBMC but not in THP-1, in a concentration-dependent mode. Probiotics were shown to play a major role, but β-glucans and elderberry extract exerted a synergistic activity. This work demonstrated that combining selected probiotics with other natural products having immunomodulatory properties is an interesting strategy to develop innovative formulations in the sector of food supplements.

## 1. Introduction

Immune homeostasis maintenance is the fundamental condition for a rapid and effective response induced by elements processed as “non self” [1]. Some pathological conditions and iatrogenic causes are linked to immune impairment; even in physiological conditions, however, common situations may negatively impact the immune response: psycho-physical stress, gut flora dysregulations, low temperatures, increased exposure to viral, bacterial and fungal threats [2]. Strengthening the immune system is always a difficult challenge for pharmacotherapy, intrinsically linked to the complex network of cellular and molecular interactions that are triggered.

The ATC code (Anatomical, Therapeutic and Chemical Classification) reports immune system disorders as belonging to subgroup L03. The drugs in this category are biological recombinant immunostimulants, such as colony-stimulating factors, interferons and interleukins, bacterial lysates which exert a general increase of innate and adaptive response [3] and few low molecular weight molecules, such as pidotimod, effective in upregulating antimicrobial and immunomodulatory proteins as well as increasing Toll like Receptors 2 and 4 (TLR2 and TLR4) mediated response [4]. Some medicinal plants are also officially recognized to strengthen immune response. The most important and adequately studied herbal drugs recognized by the European Medicines Agency (EMA) with immunomodulatory activity, used above all in the prevention and/or treatment of common cold, are *Echinacea purpurea* (L.) Moench. root and whole plant, *E. pallida* Nutt. root*, E. angustifolia* DC. root, *Pelargonium sidoides* DC. root preparations; *Viscum album* L. is a medicinal plant mostly used to stimulate immunity in cancer patients [5]. The interest in herbal products with immunomodulatory activity is growing, due to the multitarget action exerted by some phytocomplexes, not only in the pharmaceutical context, but also in that of food supplements [6]. Beside phytotherapy, probiotics are among the natural products most used in the sector of food supplements for the immune system because of their safety of use and efficacy, which has been reported in several clinical trials and in meta-analysis as well [7,8]. Several bacterial strains, capable of reaching the gut alive and of interact in a favorable manner with the microbiota have been selected and are currently used, among those, *Bifidobacterium* spp. and *Lactobacillus* spp. are the most known probiotics [9].

This sector of food supplements is growing more and more, year by year, pushed by a favorable consumers’ perception oriented towards health maintenance, but also because of high innovation in research and development which has allowed for the introduction of new ingredients in the market, pharmaceutical-like forms for oral use with improved pharmakokinetic characteristics and new combinations of ingredients [6,7,8,9,10,11]. 

However, testing food supplements is challenging for scientific community because the target population of consumers is healthy people and the thin difference between pathology and physiological dysregulation should be addressed; therefore, collecting data on actual healthy functions of food supplements is not usual and the most part of products are in the market without a proper scientific validation [6]. 

In vitro models may help in understanding cell and molecular modulations and allow to compare complex combinations and single ingredients, as well as reference drugs, in order to have a quali-quantitative comparison of a definite effect. 

Recently, our group focused his attention on some marketed associations between probiotics and herbal extracts and we were able to demonstrate the protective effect of a fixed combination of probiotics and herbal extracts in an in vitro co-culture model of intestinal inflammation, by regulating inflammatory markers of impaired intestinal barrier function [12].

With the aim of investigating the biological effect of two new food supplements marketed within the European Union (EU), in this work we investigated for the first time the mode of action of two combinations of selected probiotic strains with botanical extracts thought to maintain a healthy immune system in adults and children. The formulation for adults is a combination of a probiotic (*Bifidobacterium animalis* subsp*. lactis* Bl-04^®^, isolated from human intestine) with a yeast derivative (1,3-1,6-β-glucans from *Saccharomices cerevisiae*); the formulation for children is an association between *Lactobacillus rhamnosus* CRL1505 (isolated from goat milk) and elderberry extract. The two formulations combine specific probiotic strains, genetically characterized and pre-clinically and clinically tested for immune regulation [13,14], associated with botanical functional ingredients also claimed for their activity on immune system [15]; innovation and rationale of the formulations are witnessed by the release of a patent for their industrial invention [16]. 

Bearing in mind the complexity of tested formulations, in this very first study we aimed to monitor the cellular downstream effect of these combinations on immune response by dosing different cytokines expressed at different times, both in ex vivo human peripheral blood mono-nuclear cells (PBMC) and in THP-1 human monocytes. To complete the work, we compared the effect of the two formulations with single ingredients, with a pure pro-inflammatory reference, bacterial lipopolysaccharide (LPS), and with a reference immunomodulatory drug, namely pidotimod.

## 2. Materials and Methods

### 2.1. Tested Samples and Probiotics Preparation

The two tested formulations are composed as reported in Table 1.

All the ingredients and finished formulations were supplied by Schwabe Pharma Italia (Egna, Bozen, Italy).

Probiotics of both formulations were activated in peptone water: 20 mg of *Bifidobacterium animalis* subsp*. lactis* Bl-04^®^ and *Lactobacillus rhamnosus* CRL1505 (lyophylized powder, >5 × 10^8^ CFU/mg), as well as 500 mg of BLBG and LRSN were dissolved in 20 mL of peptone water and left at 37 °C for 24 h. The probiotics viability was visually inspected after classical bacterial agar sub-culture of an aliquot of both samples [17]. 

Peptone water was prepared, as per supplier indication (Sigma-Aldrich, Milan, Italy), by dissolving 25 g of casein peptone/soylecithin broth (Sigma-Aldrich) in 960 mL distilled water, heated at 50 °C for approximately 30 min, until completely dissolved. Then, 40 mL of polysorbate (Tween^®^ 20) (10% *v*/*v* in water) was added and the mixture, regulated to 1 L, was sterilized by autoclaving at 121 °C for 15 min. 

After probiotics reactivation, samples were filtered 0.22 μm. 

Peptone water was also used to prepare β-glucans and elderberry extract solutions.

### 2.2. THP-1 and Peripheral Blood Mononuclear Cells (PBMC) Culture

THP-1 cells were cultured in 75-cm^2^ flasks with RPMI 1640 medium containing 10% *m*/*v* fetal bovine serum (FBS) (Euroclone, Milan, Italy), 1% *m*/*v* L-glutamine, 1% *m*/*v* antibiotic (penicillin and streptomycin, Sigma-Aldrich) and 0.05% *m*/*v* 2-mercaptoethanol (Sigma-Aldrich). The flasks were incubated at 37 °C in CO_2_-enriched humid atmosphere (5%) with a daily medium change until cell confluence of 70%. EDTA-trypsin solution (Sigma-Aldrich) was used to detach cells from the flasks. Cell counts were performed microscopically (Leica, Wetzlar, Germany, 25×) using a hemocytometer [18]. 

According to the permission for using own blood for only scientific and non-diagnostic purposes as declared in and according to [19,20], peripheral blood mononuclear cells (PBMC) were isolated from the blood of GC and GB by density gradient centrifugation, using Histopaque^®^-1077 (Sigma-Aldrich). The PBMC ring was recovered, washed and suspended in a culture medium consisting of RPMI 1460 medium with 1% L-glutamine and 1% penicillin/streptomycin solution. Cell counting was performed using a hemocytometer and Trypan blue staining.

### 2.3. Treatments 

In 24-wells plates, PBMC and THP-1 cells were seeded in RPMI 1640 medium supplemented with antibiotics and glutamine (and FBS 3% for THP-1), at a density of 1 × 10^6^ and 5 × 10^4^ in 1 mL, respectively. PBMCs were immediately treated, while THP-1 were incubated at 37 °C and 5% CO_2_ for 24 h before treatments. In triplicate, both RPMI and peptone water at the same maximum concentration used in diluted samples (1:1000) were placed in the control wells (CTRL), while samples at different concentrations were placed in treatment cell groups. In detail: BLBG and LRSN were tested at 25-5-1 μg/mL of the whole formulation, where active ingredients were all <20 μg/mL as suggested by [21] in order to proof immunomodulatory agents in vitro. β-glucans and *Sambucus nigra* L. glyceric extract were also studied alone at 25 μg/mL and at concentrations present in formulations. Probiotics of BLBG and LRSN, namely *Bifidobacterium animalis* subsp*. lactis* Bl-04^®^ and *Lactobacillus rhamnosus* CRL1505, after activation, were used diluting the peptone broth 1000-fold to obtain a concentration of starting powder 1 μg/mL. Pidotimod was used at 5 μg/mL, lipopolysaccharide (LPS) from *Salmonella enteriditis* (Sigma-Aldrich ref. L8643-10 mg) at 100 ng/mL.

Samples were tested at two different timings, 6 and 24 h, short and long exposure times that simulate a short term and chronic intake and, more important, that cover the whole curve of maximum release of the most part of cytokines as previously verified by our group in different cell lines [12,13,14,15,16,17,18,19,20] and as reported in [22] for THP-1 and [23,24] for PBMC.

Two experiments with a technical triplicate (n = 6) were performed. 

### 2.4. Cytokine Dosage

Non-competitive sandwich type ELISA kits were used for the assays.

TNF-α (Thermo Fisher, Waltham, MA, USA, ref. 88-7346-22), IL-2 (Thermo Fisher, 88-7025-22), IL-6 (Thermo Fisher, 88-7066-22), IL-10 (Thermo Fisher, 88-7106-22), IFN-γ (Thermo Fisher, 88-7316-22) were dosed in PBMC, whereas TNF-α, IL-6, IL-8 (Thermo Fisher, 88-8086-22) were dosed in THP-1. 

Treated and not treated cells underwent three freezing/thawing cycles: −80 °C/+20 °C and ELISA dosages were performed according to the data sheets provided by the supplier. The plate reader used was a Perkin Elmer Nivo 3s (Waltham, MA, USA).

### 2.5. Statistical Analysis

The statistical difference of results between different experimental groups is determined by analysis of variance (ANOVA) followed by T student tests. Statistically significant values are those with *p* < 0.05 when samples and controls are compared. Graphs and calculations are made using the GraphPrism^®^ program and Microsoft Excel 2019.

## 3. Results

**Figure 1:**
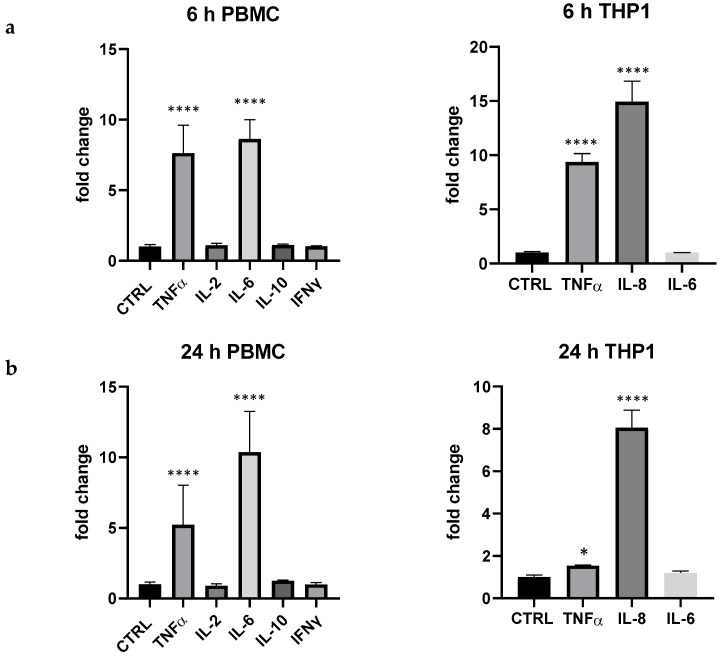
Cytokine expression produced by LPS 100 ng/mL in PBMC and THP-1 after 6 (**a**) and 24 h (**b**) of treatment. Values are normalized to control and expressed as fold change. *: *p* < 0.05 vs. ctrl; ****: *p* < 0.0001 vs. ctrl (one-wayANOVA).

The experimental design was validated when data on LPS 100 ng/mL were recorded; in fact, in PBMC, even at very low concentration, the endotoxin produced a marked and rapid upregulation of TNF-α and IL-6, an increase of IL-10, even if not statistically different from the control; IL-2 and IFN-γ were not upregulated. TNF-α, IL-8, but not IL-6 were upregulated in THP-1 (Figure 1). 

**Figure 2:**
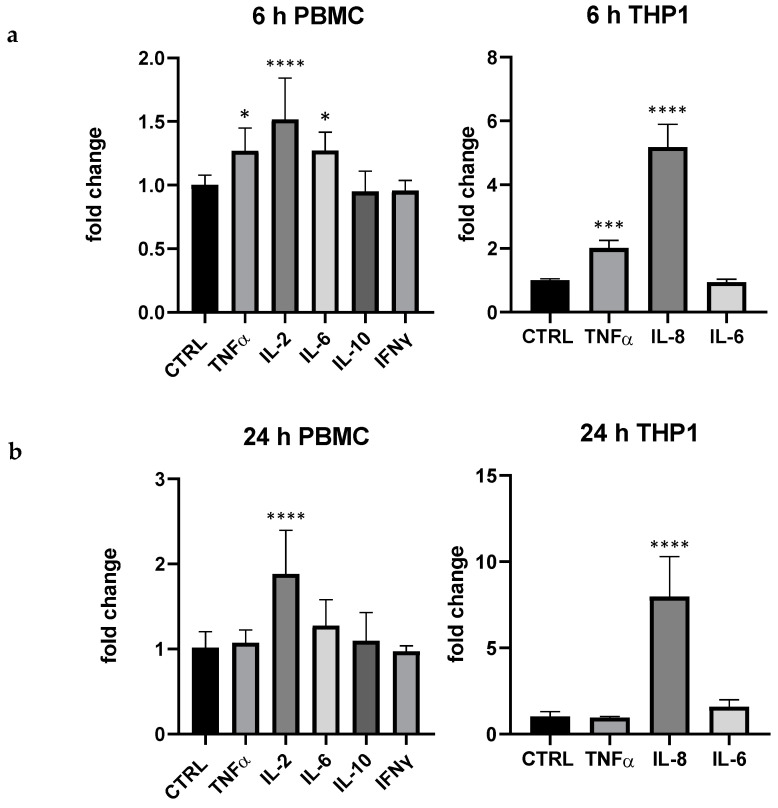
Effect of pidotimod (5 μg/mL) on cytokine expression produced by PBMC and THP-1 at 6 h (**a**) and 24 (**b**). Values are normalized to control and expressed as fold change. *: *p* < 0.05 vs. ctrl; ***: *p* < 0.001 ****: *p* < 0.0001 vs. ctrl (one-wayANOVA).

In comparison with LPS, pidotimod 5 μg/mL exerted a quantitative effect clearly different: interestingly, in THP-1 pidotimod highly modulated IL-8 (more than 7 folds at 24 h) and, even if in a lesser extent, TNF-α at 6 h (+95% compared to control), whereas in PBMC the immunostimulant drug mainly modulated the lymphocyte activity regulator IL-2 (6 and 24 h, respectively +52 and +88%); in PBMC also TNF-α and IL-6 were upregulated at 6 h, +27% for both compared to the control, respectively (Figure 2). 

**Figure 3:**
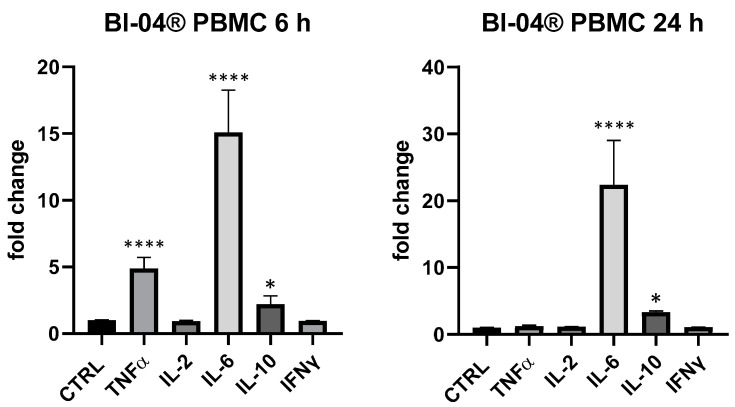
Cytokines expression produced by the bifidobacterium *B. lactis* subsp. *animalis* Bl-04^®^ in PBMC after 6 and 24 h. Values expressed as fold change. *: *p* < 0.05 vs. ctrl; ****: *p* < 0.0001 vs. ctrl (one-wayANOVA).

In regard to selected probiotic strains, in this work we wanted to evaluate the biological response of *B. animalis* subsp*. lactis* Bl-04^®^ and *L. rhamnosus* CRL1505 in this simple but effective model of cytokines expression in PBMC and THP-1, confirming that probiotics were able to upregulate cytokines expression, in particular in PBMC. In this ex vivo mixed cell line, *B. animalis* subsp*. lactis* Bl-04^®^ produced a huge upregulation of IL-6 (6 and 24 h), a significant upregulation of TNF-α (+386% at 6 h) and IL-10 (+121% at 6 h and +220% at 24 h) and only a very low modulation of IL-2 at 24 h (+14% vs. *p* > 0.05) (Figure 3). In THP-1 cells *B. animalis* subsp*. lactis* Bl-04^®^ produced only an upregulation of IL-8 at 6 h and IL-6 at 24 h (+23% and +20%, respectively, *p* < 0.05).

**Figure 4:**
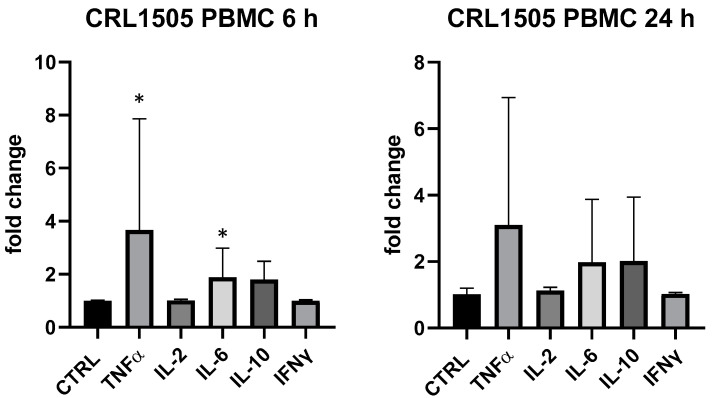
Cytokines expression produced by *Lactobacillus rhamnosus* CRL1505 in PBMC after 6 and 24 h. Values expressed as fold change. *: *p* < 0.05 vs. ctrl (one-wayANOVA).

Conversely, *L. rhamnosus* CRL1505 produced a very variable response in PBMC in different replicates, but it mainly upregulated TNF-α (+367% vs. ctrl at 6 h, *p* < 0.05) and IL-6 (+88% vs. ctrl at 6 h, *p* < 0.05); TNF-α, IL-6 and IL-10 were upregulated (about 2-fold) at 24 h but statistical analysis returned a *p* value > 0.05 (Figure 4). In THP-1 *L. rhamnosus* CRL1505 was devoid of activity.

After having effectively discriminated the effect of a pro-inflammatory agent (LPS), an immunostimulant drug (pidotimod) and the two patented probiotic strains studied as single compounds, the attention of this study was focused on the two combinations of probiotics and natural substances BLBG and LRSN. 

A very important note for interpreting results in the proper way: in both formulation probiotics are contained as much as 1 × 10^9^ CFU/dose (2 mg in 260 mg of the whole formulation in BLBG and 2 mg in 400 mg ca. in LRSN), therefore the dilution of the whole formulation, initially dissolved in peptone broth at 25 mg/mL, to the final concentration 1, 5 and 25 μg/mL, produced a drastic lower viable content of cultured CFU compared to probiotics cultured alone (1 μg/mL). Actually, this was the scope of our comparison, as at the best of our expertise it was impossible to standardize the growth curve of *B. animalis* subsp*. lactis* Bl-04^®^ and *L. rhamnosus* CRL1505 alone and in combination with other ingredients. For this reason, we chose to test BLBG and LRSN, excluding a higher content of viable CFU compared to probiotics tested as single ingredient. 

**Figure 5:**
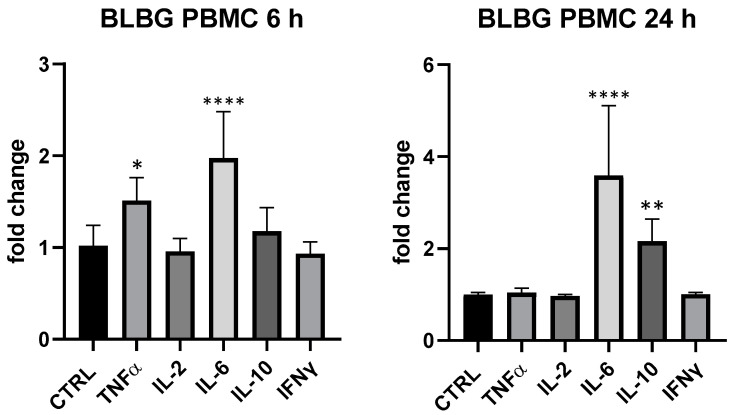
Immunomodulatory effect of the formulation BLBG, composed by *B. lactis* subsp. *animalis* Bl-04^®^ and *β*-gliucans: cytokines expression in ex vivo human PBMC at 6 and 24 h. Values expressed as fold change. *: *p* < 0.05 vs. ctrl; **: *p* < 0.01 vs. ctrl; ****: *p* < 0.0001 vs. ctrl (one-wayANOVA).

BLBG showed to upregulate TNF-α and IL-6 expression at 6 h and IL-6 and IL-10 after 24 h on PBMCs, in perfect coherence with the release kinetics of these mediators and in accordance with data recorded on *B. animalis* subsp*. lactis* Bl-04^®^. The formulation showed a concentration-dependent effect and the maximum effect was obtained at 25 µg/mL: IL-6 (6 and 24 h) and IL-10 (24 h) resulted the most upregulated cytokines compared to control (IL-10 + 116% vs. ctrl, *p* < 0.01 and IL-6 + 97% at 6 h and +259% at 24 h compared to ctrl, *p* < 0.0001); at 6 h also TNF-α was modulated in a significant manner in comparison with control of not treated cells (+51% vs. ctrl 6 h, *p* < 0.05) (Figure 5). At 5 µg/mL, only a slight upregulation of IL-6 (+42.6% vs. ctrl, *p* > 0.05) could be observed for BLBG and no modulation was observed at 1 µg/mL. 

The simultaneous regulation of TNF-α, IL-6 and IL-10 in PBMCs, where the dominant sub-population is the T helper lymphocyte, suggests a clear involvement of these leukocytes in the modulatory activity on cytokines expression exerted by BLBG; it was arguably the contribution of monocytes as well, as these cells have the ability to produce the same cytokines and to drive T helper cells towards Th1, Th2 or Treg polarization. The non-modulation of IFNγ and IL-2 and the upregulation of IL-10 also suggested an immune driving towards the Th2 and Treg produced by BLBG. 

In contrast, BLBG did not show to modify cytokine release in THP-1, confirming the important cross-talk between lymphocytes and monocytes in the formulation-induced effect. 

To better understand the role of the individual constituents of the formulation, the concentration/efficacy relationships of β-glucans was investigated.

**Figure 6:**
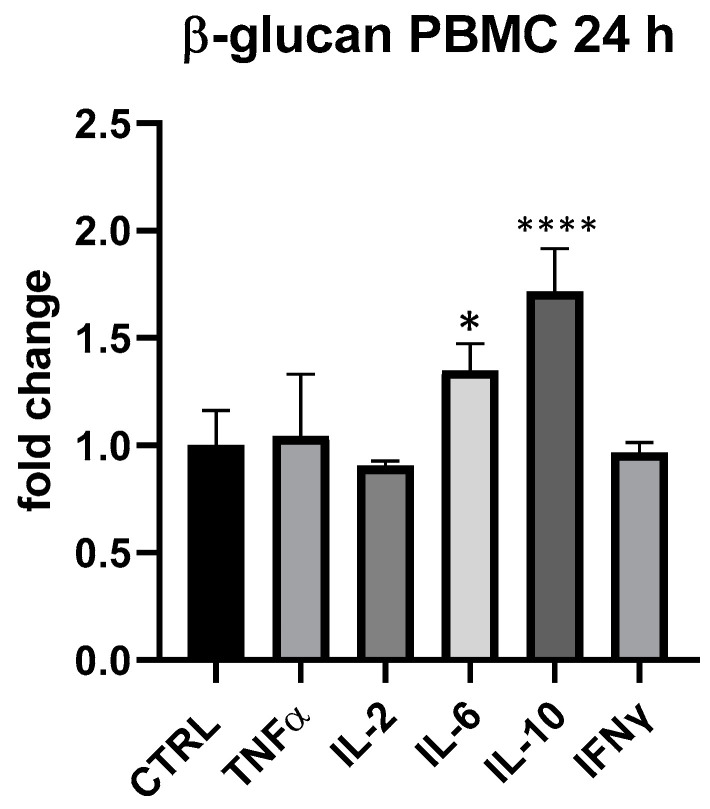
Cytokines expression produced by β-glucan from *S. cerevisiae* in PBMC after 24 h of treatment. Values expressed as fold change. *: *p* < 0.05 vs. ctrl; ****: *p* < 0.0001 vs. ctrl (one-wayANOVA).

Τhe results obtained in PBMC cell groups treated with β-glucans (25 and 18.75 μg/mL) provided evidence of their immunogenic importance since the average expression of IL-6 and IL-10 was always higher than in control. At 18.75 μg/mL, β-glucans slightly upregulated IL-6, but differences compared to control were not statistically significant. On the other hand, at 25 μg/mL, especially at 24 h, IL-6 and IL-10 upregulation was significant, as shown in Figure 6: IL-6 + 35% (*p* < 0.05) and IL-10 + 72% (*p* < 0.0001). On THP-1, β-glucans provided a very modest impact, in line with what was seen for BLBG. 

The differential evaluation of BLBG and its single ingredients allowed us to understand the important immunomodulatory role of both probiotic and β-glucans that are likely to converge in the capacity of upregulate the expression of IL-6 and IL-10 in PBMC.

**Figure 7:**
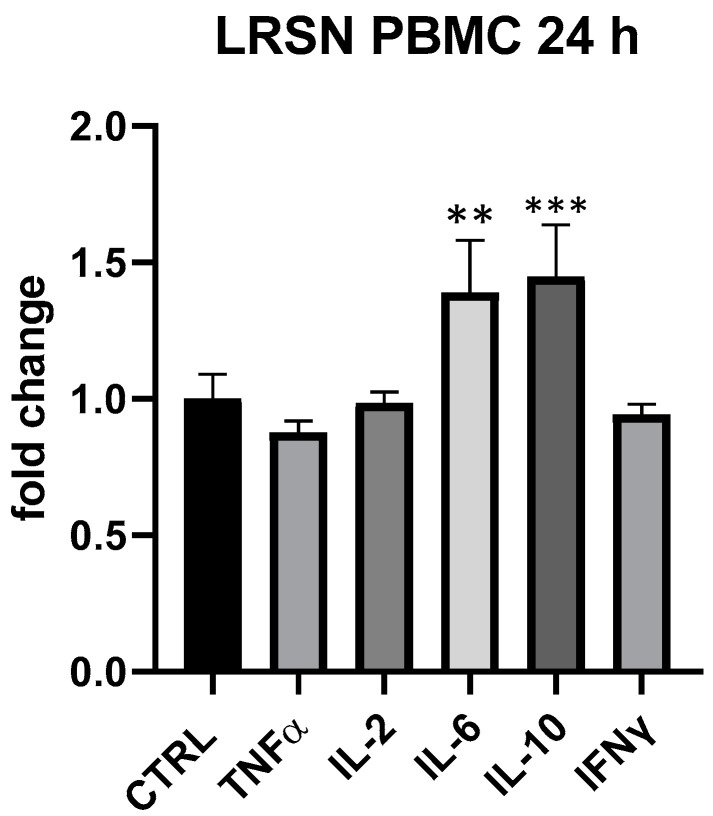
Immunomodulatory effect of the formulation LRSN, composed by *L. rhamnosus* CRL1505 and elderberry flowers and fruit extract. Values expressed as fold change. **: *p* < 0.01 vs. ctrl; ***: *p* < 0.001 vs. ctrl (one-wayANOVA).

LRSN showed to modulate in a specific way IL-6 and IL-10 at all tested concentrations in PBMC, even if only at 25 μg/mL and 24 h differences between treated and control cells were statistically significant (IL-6 + 39%, *p* < 0.01, IL-10 + 45%, *p* < 0.001 vs. ctrl) (Figure 7). In THP-1 the formulation was devoid of any effect. Here again, therefore, it was plausible a primary modulation of Th-2 and T-reg subpopulation. Interestingly, at 5 but not at 25 μg/mL LRSN modulated TNF-α even if differences in comparison to control were not statistically significant. As observed for BLBG, LRSN also did not modulate cytokines expression in THP-1.

**Figure 8:**
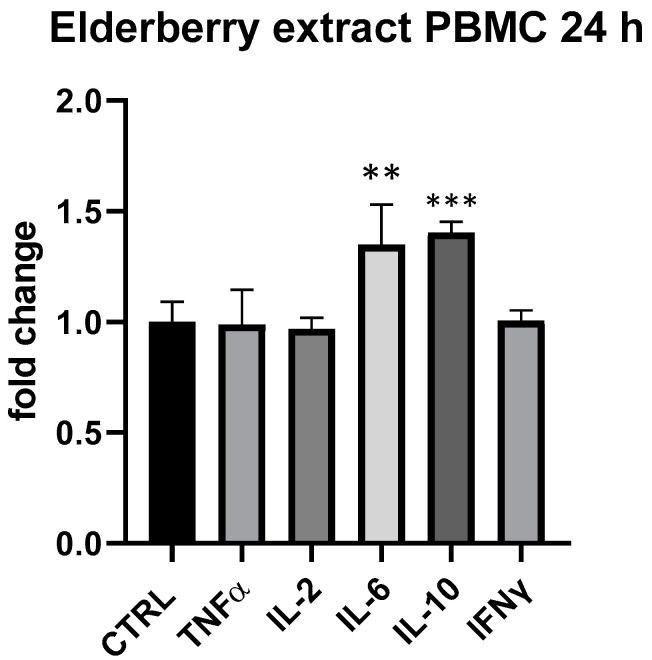
Cytokines expression produced by elderberry flowers and fruit extract in PBMC after 24 h of treatment. Values expressed as fold change. **: *p* < 0.01 vs. ctrl; ***: *p* < 0.001 vs. ctrl (one-wayANOVA).

In regard to the individual constituents, elderberry extract showed a slight modulatory effect on cytokine expressed by PBMCs at 25 μg/mL at 24 h. In particular, similarly to β-glucans, IL-6 and IL-10 were upregulated by elderberry extract (IL-6 + 35%, *p* < 0.01, IL-10 + 40%, *p* < 0.001, compared to ctrl.) (Figure 8). 

## 4. Discussion

Maintaining health is challenging for everyone and one of the most important goals is to prevent immunity impairment, in order to rely on effective natural defenses against different pathogens, but also to guarantee metabolic homeostasis and avoid DNA damage and cell cycle abnormalities [25]. The general concept of an immunity boost is not applicable in medicine, because of very individual needs but, actually, paying more attention to some vitamins and micronutrients consumptions may be helpful in preserving immune cell functions [26]. Immune system health is also intrinsically related to gut health and eubiosis [27], and this is the reason that the use of probiotics has a strong rationale in gut microbiota disturbances. The scientific research on natural products for the immune system is very topical and debated and allows understanding in a clearer way the role of many fungal compounds and herbal preparations from medicinal plants that, through different mechanisms including direct interaction with immune cell surface receptors, or intracellular pathways modulation, until an aspecific antioxidant activity, may exert an immunomodulatory effect [28]. In many food supplements, marketed in Europe as well as in other countries worldwide, the evidence aforementioned is exploited to formulate new products.

With the aim of investigating the healthy potential of these products in a scientific manner, in this work we studied two patented formulations, one developed for adults and one for children, composed by a fixed combination of selected probiotics and β-glucans or elderberry extract, respectively. Although the complexity of these formulations made it difficult to investigate the exact mechanism of action, it was still possible to make a downstream assessment of the final cellular response, in this specific case represented by cytokines expression, a parameter used both in pre-clinical and also clinical studies [29]. We chose to test the formulations both on human THP-1 and ex vivo PBMC at two different times of exposure, in order to discriminate the different involvement of monocytes and lymphocytes in the mode of action of tested samples and the expression time course of different cytokines. This simple but validated model allowed us to compare whole formulations with their single ingredients and also with the inflammatory stimulus LPS and with the drug pidotimod. We focused our attention on PBMC because, even if a large variability in the effect produced by a substance is actually expected when different cell samples are used, it is indubitable that currently this represents the most physiological model to investigate the effect of an immunomodulatory agent. 

Results obtained by means of cytokines dosage allowed us to clearly distinguish the effect of different products and the effect of LPS made immediately understandable the rapidity and the power of a pro-inflammatory stimulus which trigger cell surface receptors such as TLR2/4 [30]. On the other hand, pidotimod, even at low concentrations, confirmed to regulate both monocytes and the whole complex of PBMC where dendritic cells, which are the main target of the drug, are also present. [31] Interestingly, pidotimod primarily modulated the two characteristic effectors for monocytes and lymphocytes activation, IL-8 and IL-2, as previously reported [32]. 

Evidence suggests that probiotics act on the immune system by directly targeting immune cells through some known mechanism that include the upregulation of TLRs, in particular TLR-2, and the modulation of transduction signals [33,34]. Actually, our data demonstrated that also at low concentrations, *B. animalis* subsp*. lactis* or *L. rhamnosus* immunogenic components produced a strong response, in particular in PBMC, exerting a rapid upregulation of TNF-α, IL-6 and IL-10, even if completely different from LPS. Preliminarily, to assess the experimental protocol, we proved the activity of probiotics, testing them at higher concentration starting from a richer culture in peptone broth and obtaining an increase in cytokines release both in PBMC and in THP-1 (data not shown).

Previously published papers on in vitro investigations on *Bifidobacterium* and *Lactobacillus* spp. reported data in large part in accordance with those obtained in this work. López et al. [35] reported that many tested *Bifidobacterium* strains were able to activate dendritic cells maturation and to promote cytokines production in PBMC even if in different manner depending on diverse strains: *B. animalis* subsp. *lactis* strains enhanced IFN-γ, TNF-α and IL-10, but not IL-2 or IL-17. In this cited work, bacteria were UV inactivated but their density in final cell medium was higher than in our work, as witnessed by a similar response produced by tested strains and LPS. Interestingly, the work of Fong et al. [36] which tested *Lactobacillus rhamnosus* GG in PBMC derived antigen-presenting cells (APCs) subtypes, furtherly reported that intact bacteria have a higher capacity in polarizing Th1 response in comparison with soluble filtered immunogenic bacterial components. 

In regard to the selected *L. rhamnosus* CRL1505, a previous research [37] has reported that this strain (high concentration of intact bacteria), similarly to *L. rhamnosus* CRL1506, in porcine intestinal epithelial cells and in porcine intestine derived adherent APCs, produced the upregulation of mRNA coding of IFN, TNF-α and IL-6 in intestinal cells and IL-1β, IFN-γ, IL-2, IL-12, TNF-α, IL-6 and IL-10 in APCs accompanied by a strong upregulation of antigen presenting and co-stimulatory molecules MHC-II and CD80/86 as well as an improved Th1 response triggered by TLR2; IL-1β, IFN-γ, TNF-α, IL-6 and IL-10 expression was upregulated as well. 

Our data supported the evidence that, also at low concentrations, immunogenic components of *B. animalis* subsp. *lactis* Bl-04^®^ and of *L. rhamnosus* CRL1505 primarily upregulate Th activity and promote TNF-α, IL-6 and IL-10 production. 

The study of the two whole patented formulations here named BLBG (for adults, containing *B. animalis* subsp. *lactis* Bl-04^®^ and β-glucans) and LRSN (for children, containing *L. rhamnosus* CRL1505 and elderberry flower and fruit extract) showed that it is possible to strengthen the activity of probiotics, even at a low physiological dosage, by combining them with other natural products that reinforce their immunomodulatory activity. Here, the β-glucans and elderberry extract demonstrated effectiveness in upregulating cytokines production in PBMC but, interestingly, the same capacity on regulating specifically IL-6 and IL-10 expression was likely synergistic with probiotics in these tested formulations.

The immunomodulatory activity of β-glucans from *S. cerevisiae* has been described in several clinical trials, but also adequately characterized in preclinical papers and in vitro investigations where the capacity of pure 1,3-1,6-β-glucans at concentration ≥ 10 μg/mL in increasing Th1, Th2 and Treg response in different immunocompetent cell lines [38,39] emerged. 

Elderberry (*S. nigra*) is a medicinal plant known for its preventive activity against respiratory infections, monographed for this use as a traditional herbal medicinal product (THMP) by the European Medicines Agency (EMA). The immunomodulatory activity of elderberry extracts is generally ascribed to flavonoids such as isoquercitrin [40], but new evidence underlined the importance of polysaccharides in enhancing dendritic cell mediated Th cells response and increasing TNF-α, IL-6 and IFN-γ expression [41], in large part in agreement with that obtained in our study testing a polar elderberry extract.

Summarizing, the findings here obtained in this research on two formulations proposed as food supplements for the immune system confirmed the role of authorized and selected probiotic strains to target immunity cells response in a peculiar way, different from a drug and from a pro-inflammatory stimulus. At the same time, they suggested the importance of developing formulations that may exert a synergistic an improved effect.

We are clearly aware that this research has some important limits that we want to underline: this is just the first investigation on these formulations that allowed us to clarify the effect in cytokines production in different cell lines, but the investigation of molecular targets, as well as involved signaling, is far to be elucidated and it will be planned as next step. 

Nevertheless, we believe to have reached interesting findings, such as having demonstrated that simple but validated models may be used to validate formulations for their biological activity in the sector of food supplements. 

## 5. Conclusions

The research and development in the sector of food supplements for immune system made of natural products is very active currently but, too often, formulations are not experimentally validated. 

In this work, we demonstrated that combining selected probiotics with other natural products having immunomodulatory effect may lead to distinct immune cell responses; indeed, the combination of *B. animalis* subsp. *lactis* Bl-04^®^ and β-glucans and that of *L. rhamnosus* CRL1505 and elderberry extract specifically upregulated IL-6 and IL-10 expression (and that of TNF-α even if in a lesser extent) in human PBMC, but they were inactive in THP-1 response. The comparative analysis of whole formulations and single ingredients showed that probiotics had a major role, but both β-glucans and elderberry extracts contributed to the observed activity.

The two formulations clearly differed from a pro-inflammatory agent, such as LPS, and also from an immunostimulant drug such as pidotimod, which is not intended for healthy people because they are not devoid of side effects. 

## Figures and Tables

**Table 1:** Composition of the two tested formulations. The formulation 1 (BLBG), for adults, is a combination of the human probiotic *Bifidobacterium animalis* subsp*. lactis* Bl-04^®^ and 1,3-1,6-β-glucans from *Saccharomices cerevisiae.* The formulation 2 (LRSN), for children, combines *Lactobacillus rhamnosus* CRL1505 and *Sambucus nigra* L. flowers and fruits glyceric extract.

Formulation 1, Adults (BLBG)	Formulation 2, Children (LRSN)
*Bifidobacterium animalis* subsp. *lactis* Bl-04^®^ (>1 × 10^9^ per dose) 1,3-1,6-β-glucans from *Saccharomices cerevisiae* (200 mg per dose) Other components present in the formula are selenium, zinc and vitamin B6.	*Lactobacillus rhamnosus* CRL1505 (>1 × 10^9^ per dose) *Sambucus nigra* L. flowers and fruits glyceric extract (300 mg per dose) Other components present in the formula are zinc, vitamin B6, B12, C and D, inulin.

## Data Availability

Data is contained within the article.
